# Lifestyle-related factors that explain disaster-induced changes in socioeconomic status and poor subjective health: a cross-sectional study from the Fukushima health management survey

**DOI:** 10.1186/s12889-017-4247-2

**Published:** 2017-04-20

**Authors:** Masato Nagai, Tetsuya Ohira, Wen Zhang, Hironori Nakano, Masaharu Maeda, Seiji Yasumura, Masafumi Abe, Fukushima Health Management Survey

**Affiliations:** 1 Radiation Medical Science Center for the Fukushima Health Management Survey, Fukushima Medical University, Fukushima, Japan; 2 Department of Epidemiology, Fukushima Medical University School of Medicine, Fukushima, Japan; 3 Department of Disaster Psychiatry, Fukushima Medical University School of Medicine, Fukushima, Japan; 4 Department of Public Health, Fukushima Medical University School of Medicine, Fukushima, Japan

**Keywords:** Socioeconomic status, Subjective health, Disaster, Lifestyle

## Abstract

**Background:**

Socioeconomic status (SES) and lifestyle-related factors are determinants of subjective health. However, changes in SES are inevitable in times of natural disaster, while lifestyle-related factors remain modifiable. The aim of this study was to use a cross-sectional approach to examine lifestyle-related factors that may attenuate the negative impact of disaster-induced changes in SES on poor subjective health.

**Methods:**

We analyzed 33,350 men and women aged 20–64 years who were living in evacuation zones due to the radiation accident in Fukushima, Japan. Disaster-induced changes in SES were defined by living arrangements and working conditions. Using Poisson regression analysis adjusted for confounders (model 1) and lifestyle-related factors as intermediate variables (model 2), we compared the prevalence ratios (PRs) of poor subjective health of participants who did not undergo disaster-induced changes in SES (did not become unemployed, income did not decrease, and living in relative’s home/own home) with that of participants who did undergo disaster-induced changes in SES (became unemployed, decreased income, or lived in an evacuation shelter, temporary housing, or rental housing/apartment). We calculated the percentage of excess risks explained by lifestyle-related factors as follows: ((PR_model 1_ − PR_model 2_)/(PR_model 1_–1)) × 100.

**Results:**

Disaster-induced changes in SES were significantly associated with poor subjective health. The PRs (95% CIs) among participants who underwent disaster-induced changes in SES were 2.02 (1.81–2.24) for men and 1.80 (1.65–1.97) for women. After adjusting for lifestyle-related factors, we found that the PRs in men and women were remarkably attenuated, decreasing to 1.56 (1.40–1.73) and 1.43 (1.31–1.55), respectively. Controlling for lifestyle-related factors resulted in PR attenuation by 45.1% (men) and 46.3% (women). Satisfaction of sleep and participation in recreation and community activity particularly contributed to this attenuation.

**Conclusions:**

While disaster-induced changes in SES are unavoidable, lifestyle-related factors have the potential to attenuate the impact of these changes on poor subjective health.

**Electronic supplementary material:**

The online version of this article (doi:10.1186/s12889-017-4247-2) contains supplementary material, which is available to authorized users.

## Background

On March 11, 2011, the Great East Japan Earthquake struck off the Pacific coast of northern Japan. The earthquake generated a tsunami, which caused a radiation accident at the Fukushima Daiichi Nuclear Power Plant and led to the evacuation of over 160,000 residents of Fukushima Prefecture. Many of the victims permanently lost their homes and were forced to live in temporary housing or shelters.

Subjective health is a recognized comprehensive index of public health for evaluating the health of a population. It has been validated as a marker for predicting various health outcomes, such as diabetes [1], myocardial infarction [2], and mortality [3], independent of other health determinants. Socioeconomic status (SES), which comprises variables such as working conditions, income, and education, is associated with subjective health [4–11], namely, low SES consistently results in poor subjective health. In addition to SES, lifestyle-related factors, such as smoking [7, 12], alcohol consumption [13], physical activity [7, 12, 13], and sleeping [14, 15], are determinant factors of subjective health. These factors are also associated with SES [16–20]. Thus, it is hypothesized that changes that lower SES induce poor subjective health through lifestyle changes.

Importantly, disasters have a substantial impact on lifestyles and SES; specifically, disaster-induced change in SES and subsequent lower subjective health are unavoidable following a disaster. However, lifestyle-related factors are still modifiable. Thus, there are opportunities to modify lifestyle factors through various recovery support efforts for disaster victims. The association between SES and subjective health has been well researched [4–11]; however, the extent to which lifestyle-related factors explain the impact of disaster-induced changes in SES on subjective health has not been examined.

The purpose of this study was to explain the mediating effects of lifestyle-related factors on the association between disaster-induced change in SES and subjective health. We conducted a cross-sectional study of 33,350 Japanese adults who lived in the evacuation zone resulting from the nuclear accident in Fukushima in 2011. This study examined: 1) the impact of disaster-induced changes in SES on poor subjective health and 2) the mediating effects of lifestyle-related factors on that impact.

## Methods

### Study participants

The data for this study were derived from the Mental Health and Lifestyle Survey, which is included in the Fukushima Health Management Survey, a detailed description of which can be found elsewhere [21]. Briefly, a self-administered questionnaire on mental health and various lifestyle habits according to age category (0–6 years, 7–15 years, and ≥16 years) was delivered in January 2012 to all residents who lived in the evacuation zones caused by the radiation accident on March 11, 2011, in Fukushima Prefecture. The evacuation zone was a government-designated area (20 km radius) around the nuclear power plant. Of the target population of 180,604 participants aged ≥16 years, 73,433 (40.7%) responded.

We excluded the following participants from this study: those who did not provide information about subjective health (*n* = 1879) or current living arrangements (*n* = 14,859), those who reported “other” for current living arrangements (*n* = 2761), and those aged <20 or ≥65 years (*n* = 20,584). We excluded the latter set of participants because this group included many participants who were not working due to their status as students or retirees. Thus, we analyzed a total of 33,350 participants (14,913 men and 18,437 women).

The study protocol was approved by the Ethics Committee of Fukushima Medical University. Participants who returned the self-administered questionnaires were considered to have consented to participate.

### Socioeconomic status

The self-administered questionnaire included questions about disaster-induced changes in SES that assessed participants’ living and working situations. Change in living arrangements was assessed through each participant’s response to the question: “How have your living arrangements changes?” The participants indicated their current residence by circling one of the following: “evacuation shelter”, “temporary housing”, “rental housing/apartment”, “relative’s home”, or “own home”. We defined participants as having a “change in living arrangements” if their response was “evacuation shelter”, “temporary housing”, or “rental housing/apartment”. Change in working conditions was assessed with the question: “Has your work situation changed as a result of the natural disaster or nuclear accident?”. If participants responded “yes”, they checked the following items as appropriate: “became unemployed” or “income has decreased”. We defined participants as having a disaster-induced change in SES if they provided any of the above responses (i.e., “evacuation shelter”, “temporary housing”, “rental housing/apartment”, “became unemployed”, or “income has decreased”).

### Subjective health

The self-administered questionnaire included a question on subjective health. Subjective health was assessed through each participant’s response to the question: “Describe your current state of health”. The participants chose one of the following answers: very good (men 5.6%; women 3.9%), good (men 18.3%; women 13.8%), normal (men 61.3%; women 66.5%), poor (men 13.4%; women 14.4%), or very poor (men 1.4%; women 1.4%). “Poor subjective health” was defined by the answers “poor” or “very poor”.

### Lifestyle-related factors

We regarded smoking, alcohol consumption, sleep, participation in recreation and community activities, and exercise as lifestyle-related factors. These factors were assessed using the following questions and associated choices: “Do you smoke cigarettes (excluding cigars and pipes)?” (“never smoke”, “quit”, or “current smoker”); “Do you drink alcohol?” (“don’t drink or only rarely (less than once/month)”, “quit”, or “drink (at least once a month)”); “Are you satisfied with the quality of your sleep over the past month (regardless of sleep duration)?” (“satisfied”, “slightly dissatisfied”, “quite dissatisfied”, or “very dissatisfied or haven’t slept at all”); “Do you participate in recreation (karaoke or gateball, etc.) and community activities (festivals, etc.)?” (“never or rarely”, “sometimes”, or “often”); and “Do you exercise regularly?” (“almost every day”, “2–4 times/week”, “once/week”, or “almost never”).

### Statistical analysis

We used Poisson regression with robust error variance to derive prevalence ratios (PRs) and 95% confidence intervals (CIs) of poor subjective health according to disaster-induced changes in SES and to adjust for potential confounding factors because the prevalence of poor subjective health was not rare (≥10%) [22]. We calculated using the SAS software package, version 9.3 (Cary, NC, USA). Participants who did not undergo disaster-induced changes in SES (i.e., “living in relative’s home” or “own home” and did not “become unemployed” and “decrease income”) were selected as the reference group. All *P* values were two-tailed, and differences at *P* < 0.05 were accepted as statistically significant. In model 1, we considered the following variables to be potential confounding factors: age (5-year categories: 20–24, 25–29…up to and including 60–64 years), history of disease (yes or no: hypertension, diabetes, hyperlipidemia, stroke, heart disease, cancer, chronic hepatitis, pneumonia, bone fracture, or thyroid disease), history of mental illness (yes or no), activities of daily living (go shopping for daily necessities: can do by myself or can’t do by myself), education (elementary school・junior high school, high school, or vocational college/ junior college or university (4 years)・graduate school), and evacuation place (Fukushima or other prefectures). In model 2, we further adjusted for the following lifestyle-related factors: smoking (never smoked, quit, or current smoker), alcohol consumption (less than once/month, quit, or at least once a month), satisfaction of sleep (satisfied, slightly dissatisfied, or complaint (quite dissatisfied, or very dissatisfied or haven’t slept at all), participation in recreation and community activity (never or rarely, sometimes, or often), and regular exercise (almost every day, 2–4 times/week, or ≤1 time/week). Additionally, we repeated the analyses for each disaster-induced change in SES (i.e., change in living arrangements, became unemployed, and decrease income).

We calculated the percentage of excess risks explained by lifestyle-related factors as follows: (PR_model 1_ − PR_model 2_)/(PR_model 1_–1)) × 100 [23].

## Results

### Characteristics by disaster-induced change in SES

The characteristics of the study participants according to disaster-induced change in SES for men and women are shown in Table 1. For both sexes, the prevalence of poor subjective health among participants with a disaster-induced change in SES showed an almost two-fold increase. Compared with participants who did not undergo disaster-induced changes in SES, participants who did undergo disaster-induced changes in SES scored lower with respect to the following factors: mean age, prevalence of evacuation to Fukushima prefecture, never smoked, satisfied sleep, often participation in recreation and community activity, disease history in women, alcohol consumption less than once a month for women, and inability at go shopping for daily necessities by myself for men.

**Table 1:** Characteristics by disaster-induced changes in socioeconomic status among 14,913 men and 18,437 women aged 20–64 years in Fukushima Health Management Survey, Fukushima, 2012

	Men			Women		
	Unchanged	Changed	*P* value^b^	Unchanged	Changed	*P* value
No. of participants	4133	10,780		5302	13,135	
Mean age (years) (SD^a^)	49.1 (12.6)	46.7 (12.8)	<0.001	48.0 (12.6)	45.1 (12.8)	<0.001
Prevalence of poor subjective health (%)	8.6	17.2	<0.001	10.0	18.1	<0.001
History of disease (%)	53.6	54.2	0.520	44.7	41.2	<0.0001
Hypertension	32.2	32.8	0.483	23.9	22.0	0.005
Diabetes	15.8	15.8	0.979	9.7	8.9	0.113
Dyslipidemia	30.7	33.1	0.005	25.9	23.8	0.003
Cancer	2.3	2.4	0.865	3.6	2.7	0.001
Stroke	2.4	2.5	0.684	1.2	1.3	0.882
Cardiac disease	5.3	5.7	0.306	3.1	3.2	0.724
Liver disease	1.8	2.2	0.108	1.3	1.1	0.206
Pneumonia	2.0	2.1	0.502	2.0	2.1	0.679
Fracture	3.7	3.2	0.160	4.3	3.5	0.009
Thyroid disease	1.2	0.8	0.032	4.7	4.4	0.412
History of mental illness (%)	4.8	4.5	0.405	4.2	5.7	<0.0001
Activities of daily living (Go shopping for daily necessities) (%)						
Can’t do by myself	1.7	1.2	0.008	1.2	1.0	0.202
Educational status (%)						
Vocational college/ junior college, university•graduate school	28.2	28.2	0.053	34.2	34.7	<0.0001
High school	54.5	56.1		51.3	54.4	
Elementary school •junior high school	17.3	15.7		14.5	11.0	
Evacuation place (%)						
Fukushima prefecture	96.3	77.2	<0.001	95.7	72.7	<0.001
Other prefectures	3.7	22.8		4.3	27.4	
Smoking (%)						
Never smoked	26.2	22.3	<0.001	81.0	71.1	<0.001
Quit	33.8	31.5		8.9	12.2	
Current smoker	39.9	46.2		10.1	16.8	
Alcohol consumption (%)						
Less than once/month	27.9	26.3	0.051	66.1	60.9	<0.001
Quit	2.8	2.5		1.6	2.4	
At least once a month	69.3	71.3		32.3	36.7	
Satisfaction of sleep (%)						
Satisfied	45.9	31.7	<0.001	34.5	24.2	<0.001
Slightly dissatisfied	44.0	47.7		52.4	51.9	
Complaint	10.2	20.6		13.1	23.9	
Participatin in recreation and community activity (%)						
Never or rarely	50.9	70.9	<0.001	59.5	75.6	<0.001
Sometimes	35.9	23.6		33.7	20.7	
Often	13.2	5.5		6.8	3.7	
Regular exercise (%)						
Almost every day	11.5	10.8	0.190	7.7	7.7	0.880
2–4 times /week	14.4	13.7		14.5	14.3	
≤1 time /week	74.1	75.6		77.8	78.1	

^a^ *SD* standard deviation

^b^ *P* values were calculated by chi-square test (categorical variables), or t-test (continuous variables)

### Disaster-induced change in SES and poor subjective health

Table 2 shows the PRs of poor subjective health due to disaster-induced changes in SES with 95% CIs. The association between disaster-induced changes in SES and poor subjective health was attenuated after adjustment for lifestyle-related factors. In model 2, the PRs (95% CIs) among participants who underwent disaster-induced changes in SES decreased from 2.02 (1.81–2.24) in men and 1.80 (1.65–1.97) in women to 1.56 (1.40–1.73) in men and 1.43 (1.31–1.55) in women (model 1). The percentage of excess risk explained was 45.1% for men and 46.3% for women.

**Table 2:** Prevalence ratios and 95% confidence intervals of poor subjective health by disaster-induced changes in socioeconomic status among 14,913 men and 18,437 women aged 20–64 years in Fukushima Health Management Survey, Fukushima, 2012

	Men		Women	
	Unchanged	Changed	Unchanged	Changed
No. of participants	4133	10,780	5302	13,135
No. of cases	354	1858	529	2383
Crude	1.00 (reference)	2.01 (1.81–2.24)	1.00 (reference)	1.82 (1.66–1.99)
Age-adjusted	1.00 (reference)	2.10 (1.88–2.34)	1.00 (reference)	1.93 (1.77–2.11)
Model 1^a^	1.00 (reference)	2.02 (1.81–2.24)	1.00 (reference)	1.80 (1.65–1.97)
Model 2^b^	1.00 (reference)	1.56 (1.40–1.73)	1.00 (reference)	1.43 (1.31–1.55)
Percentage excess risks explained	45.1%		46.3%	

^a^Model 1 was adjusted for age (5-year categories), history of diseases (hypertension, diabetes, hyperlipidemia, cancer, stroke, heart disease, chronic hepatitis, pneumoia, bone fracture, or thyroid disease), history of mental illness (yes or no), activities of daily living (go shopping for daily necessities; can do by myself or can’t do by myself), education (elementary school • junior high school, high school, or vocational college/ junior college or university • graduate school), and evacuation place (Fukushima or other prefecture)

^b^Model 2 was further adjusted Model 1 for smoking (never smoked, quit, or current smoker), alcohol consumption (less than once a month, quit, or at least once a month), satisfaction of sleep (satisfied, slightly dissatisfied, or complaint), participation in recreation and community activity (never or rarely, sometimes, or often), and regular exercise (almost every day, 2–4 times/week, or ≤1 time /week)

Additional file 1: Tables S1 and S2 show the PRs of poor subjective health according to each disaster-induced change in SES with 95% CIs. We also observed an attenuation in the impact of change in living arrangements, became unemployed, and decreased income on poor subjective health after adjusting for lifestyle-related factors in both sexes.

### Lifestyle-related factors associated with disaster-induced changes in SES and poor subjective health

To examine which items enhanced the association between disaster-induced changes in SES and poor subjective health, Table 3 shows the PRs with 95% CIs for model 1 plus each lifestyle-related factor. The impact of disaster-induced changes in SES on subjective health was attenuated after adjusting for satisfaction of sleep or participation in recreation and community activity. The PRs (95% CIs) decreased to 1.64 (1.48–1.82) and 1.84 (1.66–2.05) in men, respectively, and to 1.47 (1.35–1.61) and 1.71 (1.56–1.87) in women, respectively. The corresponding percentage of excess risk explained was 37.3 and 17.6% in men, respectively, and 41.3 and 11.3% in women, respectively. However, the adjustment for other lifestyle-related factors (smoking, alcohol consumption, and regular exercise) did not change the PRs of the association between disaster-induced changes in SES and poor subjective health.

**Table 3:** Prevalence ratios and 95% confidence intervals of poor subjective health by disaster-induced changes in socioeconomic status after adjusted for each lifestyle-related factors among 14,913 men and 18,437 women aged 20–64 years in Fukushima Health Management Survey, Fukushima, 2012

	Unchanged	Changed	Percentage of excess risk explained
Men			
No. of participants	4133	10,780	
No. of cases	354	1858	
Model 1^a^	1.00 (reference)	2.02 (1.81–2.24)	−
+ Smoking	1.00 (reference)	2.01 (1.81–2.24)	1.0%
+ Alcohol consumption	1.00 (reference)	2.02 (1.82–2.25)	0.0%
+ Satisfaction of sleep	1.00 (reference)	1.64 (1.48–1.82)	37.3%
+ Participation in recreation and community activity	1.00 (reference)	1.84 (1.66–2.05)	17.6%
+ Regular exercise	1.00 (reference)	2.02 (1.81–2.24)	0.0%
Women			
No. of participants	5302	13,135	
No. of cases	529	2383	
Model 1	1.00 (reference)	1.80 (1.65–1.97)	−
+ Smoking	1.00 (reference)	1.77 (1.64–1.96)	3.8%
+ Alcohol consumption	1.00 (reference)	1.79 (1.64–1.96)	1.3%
+ Satisfaction of sleep	1.00 (reference)	1.47 (1.35–1.61)	41.3%
+ Participation in recreation and community activity	1.00 (reference)	1.71 (1.56–1.87)	11.3%
+ Regular exercise	1.00 (reference)	1.81 (1.66–1.97)	−1.3%

^a^Model 1 was adjusted for age (5-year categories), history of diseases (hypertension, diabetes, hyperlipidemia, cancer, stroke, heart disease, chronic hepatitis, pneumoia, bone fracture, or thyroid disease), history of mental illness (yes or no), activities of daily living (go shopping for daily necessities; can do by myself or can’t do by mysefl), education (elementary school • junior high school, high school, or vocational college/ junior college or university • graduate school), and evacuation place (Fukushima or other prefecture)

Table 4 shows the impact of each lifestyle-related factor on poor subjective health. Satisfaction of sleep and participation in recreation and community activity was significantly associated with poor subjective health. Compared with participants who were satisfied with their sleep, participants who were slightly dissatisfied had PRs of 2.94 (men, 2.51–3.44) and 2.67 (women, 2.28–3.12), and those who had complaints had PRs of 8.26 (men, 7.08–9.63) and 7.63 (women, 6.55–8.89). Compared with participants who often participated in recreation and community activity, participants who sometimes participated had PRs of 1.12 (men, 0.90–1.40) and 1.28 (women, 1.01–1.63), and those who never or rarely participated had PRs of 1.65 (men, 1.34–2.03) and 1.75 (women, 1.39–2.20).

**Table 4:** Prevalence ratios and 95% confidence intervals of poor subjective health by lifestyle-related factors among 14,913 men and 18,437 women aged 20–64 years in Fukushima Health Management Survey, Fukushima, 2012

	Model2	
	Men	Women
Smoking		
Never smoked	1.00 (reference)	1.00 (reference)
Quit	1.04 (0.94–1.15)	1.07 (0.97–1.19)
Current smoker	1.01 (0.92–1.12)	1.08 (0.99–1.17)
Alcohol consumption		
Less than once a month	1.00 (reference)	1.00 (reference)
Quit	1.32 (1.13–1.56)	1.09 (0.92–1.29)
At least once a month	0.87 (0.81–0.94)	1.03 (0.96–1.10)
Satisfaction of sleep		
Satisfied	1.00 (reference)	1.00 (reference)
Slightly dissatisfied	2.94 (2.51–3.44)	2.67 (2.28–3.12)
Complaint	8.26 (7.08–9.63)	7.63 (6.55–8.89)
Participatin in recreation and community activity		
Often	1.00 (reference)	1.00 (reference)
Sometimes	1.12 (0.90–1.40)	1.28 (1.01–1.63)
Never or rarely	1.65 (1.34–2.03)	1.75 (1.39–2.20)
Regular exercise		
Almost every day	1.00 (reference)	1.00 (reference)
2–4 times /week	0.94 (0.81–1.09)	0.93 (0.81–1.07)
≤1 time /week	1.04 (0.92–1.18)	1.05 (0.93–1.18)

^a^Model 2 was adjusted for age (5-year categories), history of diseases (hypertension, diabetes, hyperlipidemia, cancer, stroke, heart disease, chronic hepatitis, pneumoia, bone fracture, or thyroid disease), history of mental illness (yes or no), activities of daily living (go shopping for daily necessities; can do by myself or can’t do by myself), education (elementary school • junior high school, high school, or vocational college/ junior college or university • graduate school), evacuation place (Fukushima or other prefecture), smoking (never smoked, quit, or current smoker), alcohol consumption (less than once a month, quit, or at least once a month), satisfaction of sleep (satisfied, slightly dissatisfied, or complaint), participation in recreation and community activity (never or rarely, sometimes, or often), regular exercise (almost every day, 2–4 times/week, or ≤1 time /week), and disaster-induced changes in SES (unchanged or changed)

While we adjusted for the effect of age, it is possible that disaster-induced changes in SES differed substantially between age groups. After stratifying by age (<40 and ≥40 years), we observed comparable results to those above (data not shown). Lifestyle-related factors that mediated the association between disaster-induced changes in SES and poor subjective health did not differ between age groups.

The above tendency was also observed with respect to the association between changed living arrangements (relative’s home/own home, rental housing/apartment, or evacuation shelter/temporary housing), became unemployed, decreased income, and poor subjective health (Additional file 1: Tables S1, S2, and S3). However, participation in recreation and community activity did not attenuate the impact of evacuation shelter/temporary housing and became unemployed on poor subjective health for women and did not attenuate the impact of decreased income for men and women.

## Discussion

Disaster-induced changes in SES (changed living arrangements, became unemployed, and decreased income) resulting from a natural disaster are associated with poor subjective health and are unavoidable. The present study yielded the following results: (1) The percentage of excess risk explained by lifestyle-related factors was 45.1% in men and 46.3% in women; and (2) The lifestyle-related factors of satisfaction of sleep and participation in recreation and community activity contributed to the attenuated association between disaster-induced changes in SES and poor subjective health.

Previous studies identified a significant association between unemployment and poor subjective health [4, 7, 9, 11]. As in previous studies, we found that became unemployed was significantly associated with poor subjective health. We also showed that decreased income and change in living arrangements were significantly associated with poor subjective health; for women, this association existed even in the absence of a decreased income. These results are in accordance with those of previous studies [10, 24]. Kennedy et al. showed that the odds ratios (ORs) of fair or poor subjective health increased with a decrease in annual income [10]. Sugimoto et al. found that the prospect that “income will decrease” non-significantly increased the risk of poor subjective health (OR, 1.31; 95% CI, 0.95–1.84) among youth, which included evacuees from the Great East Japan Earthquake [24]. Those authors also showed that an evacuation period of over 4 weeks significantly increased the risk of poor subjective health (OR, 1.44; 95% CI, 1.06–1.97).

After we adjusted for lifestyle-related factors, we found that the above associations were still statistically significant; however, the PRs were remarkably attenuated. We examined which lifestyle-related factors attenuated the association between disaster-induced changes in SES and subjective health. In both sexes, we found that the PRs decreased remarkably after adjustments for satisfaction of sleep and participation in recreation and community activity. Silva-Costa et al. showed that insufficient sleep significantly increased the risk of poor subjective health in nurses [14]. Geiger et al. showed that the ORs of poor subjective health increased with an increase in the number of days of insufficient rest/sleep [25]. Sleep problems cause various negative health outcomes, such as increased blood pressure and heart rate [26], mental health problems [27], and worse health-related quality of life [28]. In contrast, social participation has been associated with a decrease in functional disability [29] and depressive symptoms [30] and an improvement in subjective health [7, 31]. Changes in SES have reportedly caused dissatisfaction of sleep and failure to participation recreation and community activity [18, 32, 33]; those participants had lower subjective health because of their perceived worsened health condition. However, the causal relationships in the above associations are not fully understood. Thus, poor subjective health may lead to impaired quality of sleep and non-participation in recreation and community activity.

Interestingly, smoking, alcohol consumption, and regular exercise were not associated with poor subjective health in the present study. While smoking, alcohol consumption, and regular exercise are commonly recognized as determinants of subjective health [7, 11–13, 34–36], other studies have reported that smoking, alcohol consumption, regular exercise, and body mass index did not explain the impact of the log of income on subjective health [11]. Furthermore, the ORs of poor subjective health according to employment status did not change before and after adjusting for regular exercise, body mass index, and smoking [7]. The reason why smoking, alcohol consumption, and regular exercise did not mediate the impact of disaster-induced changes in SES on poor subjective health is unclear. In the present study, victims were forced to change their lifestyles as a result of the disaster; they had to become accustomed to life as evacuees and had to move repeatedly. It is possible, however, that the above lifestyle factors may not be associated with subjective health in abnormal situations such as natural disasters.

A major strength of the present study is that to the best of our knowledge, it is the first study to clarify the lifestyle-related factors that attenuate the impact of disaster-induced change in SES on poor subjective health. However, several limitations deserve consideration. First, the present study design was cross-sectional. We did not have any information on SES, lifestyles, or subjective health of the participants before the disaster. Thus, we could not confirm the causality between explanatory factors and subjective health. Lifestyle modification as an intervention does not necessarily prevent poor subjective health due to disaster-induced changes in SES. However, in the present study, the reason for the disaster-induced changes in SES was a natural disaster, followed by widespread evacuations. We argue that negative changes in lifestyle and subjective health occurred after the experience of disaster-induced changes in SES. Second, the response rate was only 40.7%. Additionally, we excluded 23.1% of the respondents aged 20–64 years. The results of this study may thus be affected by response and selection bias. However, the difference in the prevalence of poor subjective health between participants with and without this exclusion was only 0.7% point for men and 1.3% point for women. Therefore, we consider the effect of selection bias to be small. Importantly, we could not evaluate the response bias because there was no basic data on disaster-induced changes in SES, lifestyle habits, and subjective health for non-respondents. We acknowledge both of the following possibilities: participants with poor subjective health tend not to respond because they are feeling down or unwell, and/or participants with good subjective health tend not to respond because they do not have any problems. These possibilities may have led to an overestimate or underestimate of the impact of lifestyle-related factors on the associations between disaster-induced changes in SES and poor subjective health. Third, information on disaster-induced changes in SES and lifestyle-related factors was obtained from self-reported questionnaires. However, subjective health is an index that assesses subjective and self-reported. Thus, not objectively measured. For example, if people who have good sleep, as measured objectively, are dissatisfied with their own sleep, they will likely report a poor in their own subjective health. Thus, even though exposure and mediators are self-reported, modifying self-reported conditions will arguably be more important and useful than focusing on objectively measured conditions. Finally, we limited study participants to those aged 20–64 years because working conditions were used as part of the definition of disaster-induced changes in SES. Therefore, the present findings may not apply to teenagers and the elderly, and wider generalizations must be made cautiously.

## Conclusions

Our findings suggest that while SES changes are unavoidable in disaster situations, it is possible for lifestyle-related factors, especially satisfaction of sleep and participation in recreation and community activity, to attenuate the association between changes in SES due to disaster and poor subjective health. After a disaster, evacuees receive various forms of support to help them rebuild their lives. We have shown that improving satisfaction of sleep and participation in recreation and community activity through recovery support efforts may prevent the deterioration of the subjective health.

## Additional file

Additional file 1:
**Table S1.** Prevalence ratios and 95% confidence intervals of poor subjective health by change in living arrangements among 14,913 men and 18,437 women aged 20–64 years in Fukushima Health Management Survey, Fukushima, 2012. **Table S2.** Prevalence ratios and 95% confidence intervals of poor subjective health by change in working condition among 14,913 men and 18,437 women aged 20–64 years in Fukushima Health Management Survey, Fukushima, 2012. **Table S3.** Prevalence ratios and 95% confidence intervals of poor subjective health by lifestyle-related factors among 14,913 men and 18,437 women aged 20–64 years in Fukushima Health Management Survey, Fukushima, 2012. (DOCX 59 kb)

## Abbreviations

CIs: Confidence intervals
PRs: Prevalence ratios
SD: Standard deviation
SES: Socioeconomic status
