# Supplementary material for: Lifestyle-related factors that explain disaster-induced changes in socioeconomic status and poor subjective health: a cross-sectional study from the Fukushima health management survey
Source: BMC Public Health. 2017 Apr 20;17:340. doi: 10.1186/s12889-017-4247-2 (PMC5397819; doi:10.1186/s12889-017-4247-2)
Supplement: Additional file 1: — Table S1. Prevalence ratios and 95% confidence intervals of poor subjective health by change in living arrangements among 14,913 men and 18,437 women aged 20–64 years in Fukushima Health Management Survey, Fukushima, 2012. Table S2. Prevalence ratios and 95% confidence intervals of poor subjective health by change in working condition among 14,913 men and 18,437 women aged 20–64 years in Fukushima Health Management Survey, Fukushima, 2012. Table S3. Prevalence ratios and 95% confidence intervals of poor subjective health by lifestyle-related factors among 14,913 men and 18,437 women aged 20–64 years in Fukushima Health Management Survey, Fukushima, 2012. (DOCX 59 kb) [file 12889_2017_4247_MOESM1_ESM.docx]

Table S1. Prevalence ratios and 95% confidence intervals of poor subjective health by change in living arrangements

among 14,913 men and 18,437 women aged 20-64 years in Fukushima Health Management Survey, Fukushima, 2012.

Relative's home/ own home

Rental housing/ apartment

Evacuation shelter/ temporary housing

Men

No. of participants

5,882

7,135

1,896

No. of cases

594

1,227

391

Crude

1.00 (reference)

1.70 (1.55-1.87)

2.04 (1.82-2.30)

Age-adjusted

1.00 (reference)

1.86 (1.69-2.04)

2.05 (1.83-2.30)

Model 1

a

1.00 (reference)

1.80 (1.64-1.98)

1.89 (1.69-2.12)

+Smoking

1.00 (reference)

1.80 (1.64-1.98)

1.89 (1.69-2.12)

+Alcohol consumption

1.00 (reference)

1.81 (1.65-1.99)

1.87 (1.67-2.10)

+Satisfaction of sleep

1.00 (reference)

1.47 (1.34-1.60)

1.62 (1.45-1.81)

+Participatin in recreation and community activity

1.00 (reference)

1.60 (1.45-1.76)

1.78 (1.59-1.99)

+Regular exercise

1.00 (reference)

1.80 (1.64-1.98)

1.91 (1.71-2.15)

Model 2

b

1.00 (reference)

1.36 (1.24-1.49)

1.57 (1.40-1.75)

Women

No. of participants

7,101

9,246

2,090

No. of cases

779

1,666

467

Crude

1.00 (reference)

1.64 (1.52-1.78)

2.04 (1.84-2.26)

Age-adjusted

1.00 (reference)

1.83 (1.69-1.98)

2.04 (1.84-2.27)

Model 1

a

1.00 (reference)

1.71 (1.58-1.86)

1.78 (1.61-1.97)

+Smoking

1.00 (reference)

1.69 (1.56-1.84)

1.76 (1.59-1.95)

+Alcohol consumption

1.00 (reference)

1.71 (1.57-1.85)

1.77 (1.60-1.96)

+Satisfaction of sleep

1.00 (reference)

1.42 (1.32-1.54)

1.51 (1.37-1.66)

+Participatin in recreation and community activity

1.00 (reference)

1.59 (1.46-1.72)

1.79 (1.62-1.98)

+Regular exercise

1.00 (reference)

1.72 (1.58-1.86)

1.80 (1.62-1.99)

Model 2

b

1.00 (reference)

1.35 (1.25-1.46)

1.53 (1.39-1.69)

a

Model 1 was adjusted for age (5-year categories), history of diseases (hypertension, diabetes, hyperlipidemia, cancer, stroke, heart disease,

chronic hepatitis, pneumoia, bone fracture, or thyroid disease), mental illness (yes or no), activities of daily living (go shopping for daily

necessities; can do by myself or can't do by myself), education (elementary school

・

junior high school, high school, or vocational college/ junior

college or university

・

graduate school), and evacuation place (Fukushima or other prefecture).

b

Model 2 was further adjusted Model 1 for smoking (never smoked, quit, or current smoker), alcohol consumption (less than once a month, quit,

or at least once a month), satisfaction of sleep (satisfied, slightly dissatisfied, or complaint), participation in recreation and community activity

(never or rarely, sometimes, or often), and regular exercise (almost every day, 2-4 times/week, or

?

1 time /week).

Table S2. Prevalence ratios and 95% confidence intervals of poor subjective health by change in working condition

among 14,913 men and 18,437 women aged 20-64 years in Fukushima Health Management Survey, Fukushima, 2012.

No

Yes

No

Yes

Became unemployed

No. of participants

12,215

2,698

13,435

5,002

No. of cases

1,556

656

1,919

993

Crude

1.00 (reference)

1.91 (1.76-2.07)

1.00 (reference)

1.39 (1.30-1.49)

Age-adjusted

1.00 (reference)

1.85 (1.71-2.01)

1.00 (reference)

1.40 (1.30-1.50)

Model 1

a

1.00 (reference)

1.71 (1.58-1.86)

1.00 (reference)

1.36 (1.27-1.46)

+Smoking

1.00 (reference)

1.71 (1.58-1.86)

1.00 (reference)

1.34 (1.25-1.44)

+Alcohol consumption

1.00 (reference)

1.70 (1.57-1.84)

1.00 (reference)

1.36 (1.27-1.46)

+Satisfaction of sleep

1.00 (reference)

1.48 (1.37-1.60)

1.00 (reference)

1.19 (1.12-1.27)

+Participatin in recreation and community activity

1.00 (reference)

1.63 (1.50-1.76)

1.00 (reference)

1.33 (1.24-1.43)

+Regular exercise

1.00 (reference)

1.73 (1.60-1.87)

1.00 (reference)

1.37 (1.28-1.47)

Model 2

b

1.00 (reference)

1.44 (1.33-1.56)

1.00 (reference)

1.18 (1.10-1.26)

Decreased income

No. of participants

11,008

3,905

15,188

3,249

No. of cases

1,528

684

2,346

566

Crude

1.00 (reference)

1.26 (1.16-1.37)

1.00 (reference)

1.13 (1.04-1.23)

Age-adjusted

1.00 (reference)

1.25 (1.15-1.36)

1.00 (reference)

1.11 (1.02-1.20)

Model 1

1.00 (reference)

1.29 (1.19-1.39)

1.00 (reference)

1.13 (1.04-1.23)

+Smoking

1.00 (reference)

1.28 (1.18-1.39)

1.00 (reference)

1.13 (1.04-1.22)

+Alcohol consumption

1.00 (reference)

1.30 (1.20-1.41)

1.00 (reference)

1.13 (1.04-1.23)

+Satisfaction of sleep

1.00 (reference)

1.16 (1.07-1.25)

1.00 (reference)

1.04 (0.97-1.13)

+Participatin in recreation and community activity

1.00 (reference)

1.29 (1.19-1.40)

1.00 (reference)

1.14 (1.05-1.23)

+Regular exercise

1.00 (reference)

1.28 (1.18-1.39)

1.00 (reference)

1.13 (1.04-1.22)

Model 2

1.00 (reference)

1.17 (1.08-1.26)

1.00 (reference)

1.05 (0.97-1.14)

b

Model 2 was further adjusted Model 1 for smoking (never smoked, quit, or current smoker), alcohol consumption (less than once a month, quit, or at

least once a month), satisfaction of sleep (satisfied, slightly dissatisfied, or complaint), participation in recreation and community activity (never or

rarely, sometimes, or often), and regular exercise (almost every day, 2-4 times/week, or

?

1 time /week).

Men

Women

a

Model 1 was adjusted for age (5-year categories), history of diseases (hypertension, diabetes, hyperlipidemia, cancer, stroke, heart disease, chronic

hepatitis, pneumoia, bone fracture, or thyroid disease), mental illness (yes or no), activities of daily living (go shopping for daily necessities; can do

by myself or can't do by myself), education (elementary school

・

junior high school, high school, or vocational college/ junior college or university

・

graduate school), and evacuation place (Fukushima or other prefecture).

Table S3. Prevalence ratios and 95% confidence intervals of poor subjective health by lifestyle-related factors

among 14,913 men and 18,437 women aged 20-64 years in Fukushima Health Management Survey, Fukushima, 2012.

Men

Women

Men

Women

Men

Women

Smoking

Never smoked

1.00 (reference)

1.00 (reference)

1.00 (reference)

1.00 (reference)

1.00 (reference)

1.00 (reference)

Quit

1.05 (0.95-1.16)

1.07 (0.97-1.19)

1.05 (0.95-1.17)

1.08 (0.98-1.20)

1.05 (0.95-1.16)

1.08 (0.98-1.20)

Current smoker

1.01 (0.92-1.12)

1.07 (0.99-1.17)

1.02 (0.93-1.13)

1.09 (1.00-1.18)

1.03 (0.93-1.13)

1.10 (1.01-1.20)

Alcohol consumption

Less than onece a month

1.00 (reference)

1.00 (reference)

1.00 (reference)

1.00 (reference)

1.00 (reference)

1.00 (reference)

Quit

1.31 (1.11-1.54)

1.08 (0.91-1.28)

1.32 (1.12-1.56)

1.12 (0.95-1.32)

1.33 (1.13-1.57)

1.12 (0.94-1.32)

At least once a month

0.87 (0.81-0.95)

1.03 (0.97-1.10)

0.89 (0.82-0.97)

1.03 (0.96-1.10)

0.87 (0.81-0.95)

1.03 (0.96-1.10)

Satisfaction of sleep

Satisfied

1.00 (reference)

1.00 (reference)

1.00 (reference)

1.00 (reference)

1.00 (reference)

1.00 (reference)

Slightly dissatisfied

2.96 (2.53-3.46)

2.67 (2.28-3.11)

2.98 (2.55-3.49)

2.71 (2.32-3.16)

3.01 (2.58-3.53)

2.72 (2.33-3.18)

Complaint

8.34 (7.15-9.72)

7.62 (6.54-8.88)

8.43 (7.22-9.83)

7.88 (6.76-9.19)

8.64 (7.41-10.08)

8.00 (6.87-9.33)

Participatin in recreation and community activity

Often

1.00 (reference)

1.00 (reference)

1.00 (reference)

1.00 (reference)

1.00 (reference)

1.00 (reference)

Sometimes

1.10 (0.89-1.37)

1.27 (1.00-1.62)

1.15 (0.92-1.43)

1.29 (1.01-1.63)

1.16 (0.93-1.45)

1.29 (1.01-1.63)

Never or rarely

1.62 (1.32-2.00)

1.76 (1.39-2.21)

1.72 (1.40-2.12)

1.80 (1.43-2.26)

1.78 (1.45-2.19)

1.81 (1.44-2.28)

Regular exercise

Almost every day

1.00 (reference)

1.00 (reference)

1.00 (reference)

1.00 (reference)

1.00 (reference)

1.00 (reference)

2-4 times /week

0.94 (0.81-1.09)

0.93 (0.81-1.07)

0.92 (0.79-1.07)

0.93 (0.81-1.07)

0.93 (0.80-1.09)

0.93 (0.81-1.07)

?

1 time /week

1.05 (0.93-1.19)

1.05 (0.94-1.19)

1.05 (0.92-1.19)

1.04 (0.93-1.17)

1.02 (0.90-1.16)

1.03 (0.92-1.16)

Model 2

a

a

Model 2 was adjusted for age (5-year categories), history of diseases (hypertension, diabetes, hyperlipidemia, cancer, stroke, heart disease,

chronic hepatitis, pneumoia, bone fracture, or thyroid disease), mental illness (yes or no), activities of daily living(go shopping for daily

necessities; can do by myself or can't do by myself), education (elementary school

・

junior high school, high school, or vocational college/ junior

college or university

・

graduate school), evacuation place (Fukushima or other prefecture), smoking (never smoked, quit, or current smoker),

alcohol consumption (less than once a month, quit, or at least once a month), satisfaction of sleep (satisfied, slightly dissatisfied, or complaint),

participation in recreation and community activity (never or rarely, sometimes, or often), regular exercise (almost every day, 2-4 times/week,

or

?

1 time /week), and change in living arrangements (relative's home/ own home, rental housing/ apartment, or evacuation shelter/ temporary

housing), became unemployed (yes or no), or decreased income (yes or no).

Change in living arrangements

Became unemployed

Decreased income
